# Lack of Conserved miRNA Deregulation in HPV-Induced Squamous Cell Carcinomas

**DOI:** 10.3390/biom11050764

**Published:** 2021-05-20

**Authors:** Jaroslav Nunvar, Lucie Pagacova, Zuzana Vojtechova, Nayara Trevisan Doimo de Azevedo, Jana Smahelova, Martina Salakova, Ruth Tachezy

**Affiliations:** 1 Department of Genetics and Microbiology, Faculty of Science, Charles University, 12844 Prague, Czech Republic; Lucka.pagacova@seznam.cz (L.P.); zuzana.vojtechova@natur.cuni.cz (Z.V.); jana.smahelova@natur.cuni.cz (J.S.); martina.salakova@natur.cuni.cz (M.S.); ruth.tachezy@natur.cuni.cz (R.T.); 2 BIOCEV-Biotechnology and Biomedicine Centre of the Academy of Sciences and Charles University, 25250 Vestec, Czech Republic; 3 Genomics Core Facility, EMBL, 69117 Heidelberg, Germany; nayara.azevedo@embl.de

**Keywords:** squamous cell carcinoma, human papillomavirus, microRNA

## Abstract

Squamous cell carcinomas (SCCs) in the anogenital and head and neck regions are associated with high-risk types of human papillomaviruses (HR-HPV). Deregulation of miRNA expression is an important contributor to carcinogenesis. This study aimed to pinpoint commonly and uniquely deregulated miRNAs in cervical, anal, vulvar, and tonsillar tumors of viral or non-viral etiology, searching for a common set of deregulated miRNAs linked to HPV-induced carcinogenesis. RNA was extracted from tumors and nonmalignant tissues from the same locations. The miRNA expression level was determined by next-generation sequencing. Differential expression of miRNAs was calculated, and the patterns of miRNA deregulation were compared between tumors. The total of deregulated miRNAs varied between tumors of different locations by two orders of magnitude, ranging from 1 to 282. The deregulated miRNA pool was largely tumor-specific. In tumors of the same location, a low proportion of miRNAs were exclusively deregulated and no deregulated miRNA was shared by all four types of HPV-positive tumors. The most significant overlap of deregulated miRNAs was found between tumors which differed in location and HPV status (HPV-positive cervical tumors vs. HPV-negative vulvar tumors). Our results imply that HPV infection does not elicit a conserved miRNA deregulation in SCCs.

## 1. Introduction

Human papillomaviruses (HPV), species-specific viruses infecting mucosal and cutaneous epithelium of the host, can cause benign diseases such as papillomata or warts; however, some HPV types have oncogenic potential and are reported to be responsible for up to 5% of all tumors worldwide and up to 30% of those whose development is attributed to infectious agents [1]. The most common carcinoma associated with HPV infection is cervical cancer, which is caused by HPV in almost 100% of cases. HPV is also associated with other tumors of the anogenital region such as vaginal tumors, where HPV participates in 78% of cases, vulvar tumors with 25% of HPV-positive cases, anal tumors with almost 90% of HPV-positive cases, and penile tumors with 50% of HPV-positive cases [2]. The second region where HPVs play the etiological role in cancer development is head and neck, especially the oropharyngeal location [3]. The proportion of HPV-associated oropharyngeal cancers is increasing around the world, reaching 40–80% of cases in the USA and 20–90% of cases throughout Europe [4,5,6].

The proper molecular profiling of HPV-positive and HPV-negative tumors and comparisons of differences between the two etiologies is necessary for expanding our understanding of the mechanisms of carcinogenesis. There are only few publications addressing the differences in molecular profiles between HPV-associated and HPV-independent tumors. Koncar et al. determined the expression of 24 proteins by immunohistochemistry, mutations of 48 genes by sequencing, and copy number alterations for six genes by in situ hybridization for cohorts of vulvar, anal, cervical, and oropharyngeal tumors, reporting the HPV-positive tumors to have similar molecular profiles [7]. However, the HPV positivity in their samples was determined based on the p53 status, which is not an established surrogate marker for the detection of HPV. Nevertheless, Tuna and Amos compared The Cancer Genome Atlas (TCGA) database data from studies analyzing HPV-driven tumors; they found similar genomic alterations in HPV-negative and HPV-positive cases but also detected distinct epigenomic and transcriptomic profiles between these groups [8]. Genomic comparison of HPV-positive and HPV-negative oral squamous cell carcinomas (SCCs) was performed by Gillison et al. and they specified a number of unique genetic features for virus-associated tumors [9]. Similarly, significant genetic differences between HPV-positive and HPV-negative tumors were revealed in several other studies [10,11]. So far, only two studies using whole-exome sequencing of vulvar tumors with regard to HPV positivity have been published [12,13]. They have shown that genetic alterations in vulvar carcinomas encompass mutations and copy number alterations that differ between HPV-positive and HPV-negative cases, in addition to common alterations observed irrespective of the HPV status.

Promising specific, sensitive, and clinically significant biomarkers are miRNAs, small single-stranded non-coding RNAs that regulate gene expression and play an important role in cell development, growth, and differentiation. Their expression has been reported to be deregulated in tumors. MicroRNAs play a role in stimulating tumor growth by negative regulation of tumor suppressor genes or by positive regulation of oncogenes. Specific miRNA profiles have been reported in many studies of various tumors, for example, of leukemia, breast cancer, lung cancer, or prostate cancer [14,15,16,17,18]. Numerous studies focused on the analysis of miRNA profiles in cervical cancer [19,20,21,22]; however, miRNA expression in tumors of the rest of anogenital regions is less researched, with only two studies analyzing miRNA profiles in vulvar cancer [23,24]. MicroRNA expression in head and neck cancers has been increasingly studied, and several studies on cell lines or tumors have been published [25,26,27,28,29]; however, they did not examine their association with HPV status. HPV status was addressed in two studies of Lajer et al. [19,30] who defined the group of core HPV miRNAs specific for two types of HPV-positive tumors, cervical and head and neck. Furthermore, Miller et al. analyzed miRNA expression in HPV-positive and HPV-negative oropharyngeal carcinomas and validated the data against The Cancer Genome Atlas, identifying HPV-associated oncogenic miRNAs [31]. In our previous study, we focused on miRNA profiles in HPV-negative and HPV-positive tonsillar tumors and in cervical tumors and on the comparison with a model system of keratinocyte clones [32] and defined the core HPV miRNAs, but they did not overlap with those from the study by Lajer et al. The lack of comparability between the outcomes of these studies can be most likely attributed to the anatomical heterogeneity of the analyzed head and neck tumors and to the different methodological approaches.

In our study, we analyzed the miRNA expression profiles in a set of anogenital tumors associated with HPV, namely, cervical, vulvar, and anal tumors, and in a set of tonsillar tumors by next-generation sequencing (NGS) in an attempt to identify commonly deregulated miRNAs exclusive for HPV-dependent SCCs. To our knowledge, this is the first study to analyze miRNA profiles in four HPV-driven types of tumors which employs a unified methodological approach. Furthermore, this study provides miRNA expression analysis of insufficiently researched anal and vulvar carcinomas.

## 2. Materials and Methods

### 2.1. Clinical Samples

Samples of SCCs were obtained from the Department of Pathology and Molecular Medicine, 2nd Faculty of Medicine, Charles University and Motol University Hospital, Prague, and from the Institute of Pathology, 3rd Faculty of Medicine, Charles University and University Hospital Kralovske Vinohrady, Prague. All tissue samples originated from unique patients. Formalin-fixed paraffin-embedded (FFPE) samples prepared from tumor tissues were stained and macrodissected [32]. The HPV status of FFPE samples was determined by P16 immunochemistry and HPV DNA detection [33]. All HPV-positive tumors contained high-risk HPV16. For each anatomical location (tonsillar, cervical, vulvar, anal), the sample set consisted of three HPV-positive tumors, three HPV-negative tumors, and three samples of normal tissue. Cervical HPV-negative tumors were not included due to their unavailability.

### 2.2. Sample Processing for NGS

Total RNA was isolated from macrodissected sections of FFPE samples using AllPrep^®^ DNA/RNA FFPE (Qiagen, Germantown, MD, USA). RNA concentration and quality were measured by a Nanodrop^™^ spectrophotometer (Thermo Scientific, Waltham, MA, USA) and Experion chip electrophoresis (Bio-Rad, Hercules, CA, USA). From purified RNA, sequencing libraries were prepared using the TruSeq Small RNA Library prep kit (Illumina, San Diego, CA, USA). The libraries were sequenced on NextSeq 500 (Illumina, San Diego, CA, USA) at the Division of Molecular Medicine, Ruđer Bošković Institute (Zagreb, Croatia), or the EMBL’s Genomics Core Facility (GeneCore) (Heidelberg, Germany) using the NextSeq 500/550 High Output Kit (Illumina, San Diego, CA, USA). Sequencing reads were deposited in the SRA, GenBank (BioProject ID: PRJNA718204).

### 2.3. Analysis of NGS Data

Sequencing reads were trimmed off adapter sequences using the FastQ toolkit Basespace App (Illumina). The trimmed reads were mapped onto human reference genome assembly GRCh38.p13 using the Geneious mapper [34] (max. mismatches: 10%, max. gaps: 10%). The complete analysis of DE between replicate tumor samples and normal tissues (expression normalization, DE calculation, calculation of statistical significance) was carried out using the DESeq2 package [35]. The miRNAs exhibiting fold change of expression (FC) ≥ 2.0 and statistical support of *p*adj (*p*-value adjusted for multiple testing) ≤ 0.1 were extracted and further analyzed. The Adonis–Bray test [36] with 10,000 permutations was used to test the association of global miRNA expression with the sample metadata categories (tissue type, anatomic location, HPV status).

## 3. Results and Discussion

### 3.1. Global Characterization of miRNA Expression

Since deregulation of the cellular miRNA network is commonly observed in carcinogenesis, we aimed to characterize the patterns of miRNA expression in a set of tissue samples encompassing tumors from different anatomical locations and differing in HPV status. Samples of HPV-associated tumors (anogenital and tonsillar tumor tissues), of the corresponding HPV-negative tumors (if available), and of healthy tissues were collected from three patients per sample type and processed for NGS. Sequencing yields ranged from 338,068 to 4,273,955 total reads per sample; the percentage of miRNA-derived reads varied from 6.3% to 82.9% (Table S1). The expression level values were calculated for individual miRNAs as their proportion among the total miRNA reads (Table S2).

**Figure 1:**
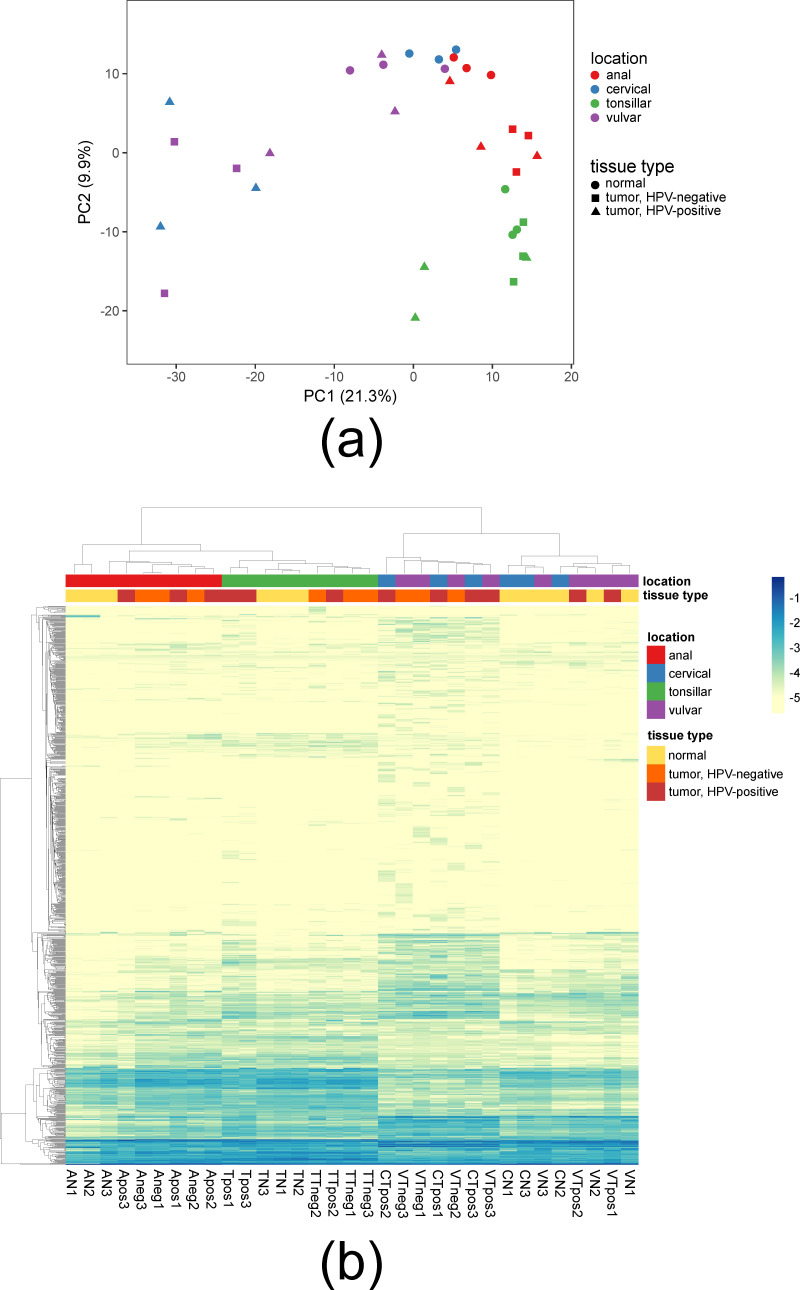
Clustering of tissue samples by miRNA expression profiles. (**a**) PCA plot; (**b**) heatmap plot. The heatmap color scale depicts log-transformed abundance values of individual miRNAs among total miRNA sequencing reads. Visualizations were carried out in ClustVis [41].

The similarity of tissue samples based on their miRNA expression profiles is visualized in Figure 1 by principal component analysis (PCA) and a heatmap. In both plots, the clustering and thus the similarity of samples correlated with their anatomical locations. Tonsillar samples formed a separated cluster. Among the anogenital samples, anal samples clustered separately from genital samples. A notable separation was detected between the genital (i.e., cervical and vulvar) samples. All cervical tumors, all vulvar HPV-negative tumors, and one vulvar HPV-positive tumor were distinct from normal cervical and vulvar tissues and two vulvar HPV-positive tumors; the extent of dissimilarity of their miRNA expression profiles was readily discernable in both PCA and the heatmap (Figure 1). The Adonis–Bray test revealed that the miRNA expression was most significantly associated with anatomical location (anal/cervical/vulvar/tonsillar; *p* = 0.0001) and tissue type (tumor/nonmalignant; *p* = 0.001) of samples. This is in accordance with miRNA expression being long recognized to exhibit tissue-specific and tumor-specific differences [37,38,39,40]. The association with HPV status (positive/negative) was weaker and not significant (*p* = 0.074).

### 3.2. Differential Expression of miRNA in SCCs

**Figure 2:**
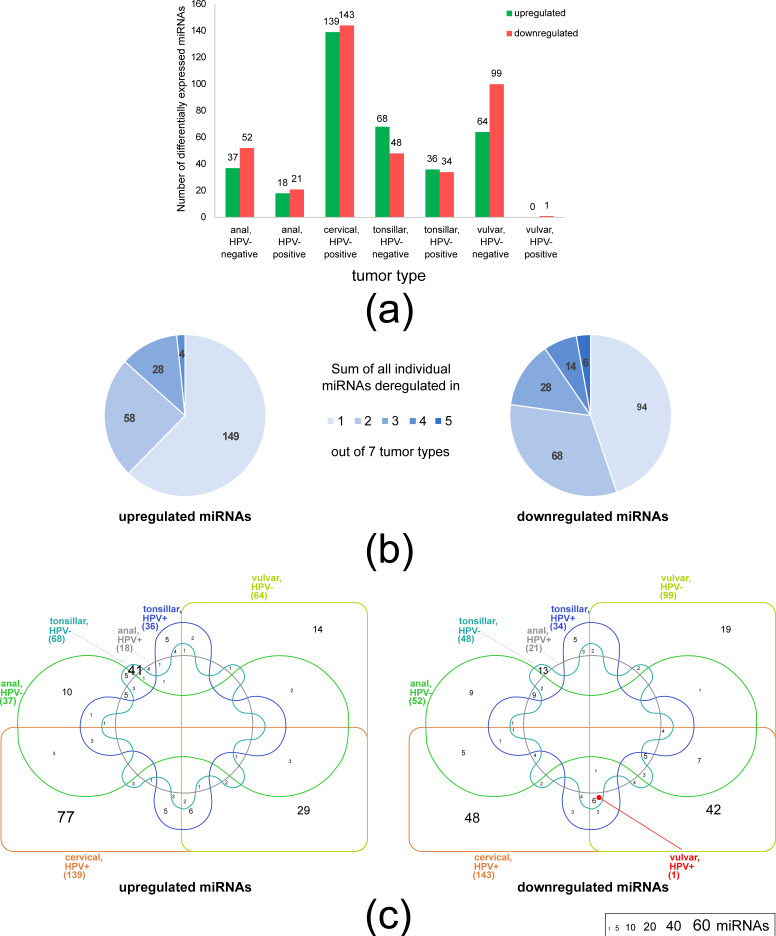
DE of miRNAs in tumors of different locations and HPV status. (**a**) Total numbers of differentially expressed miRNAs in each of the seven analyzed tumor types. (**b**) The miRNAs that exhibited shared deregulation across the seven studied tumor types. The values denote the total numbers of miRNAs which were deregulated specifically in one tumor type and those whose deregulation was common to two, three, four, or five tumor types. No miRNAs were deregulated in more than five tumor types. (**c**) Venn diagrams depicting the numbers of unique and shared differentially expressed miRNAs among the seven studied tumor types. The font size range is denoted in the legend. Zero values were omitted. The placement of the sole miRNA deregulated in HPV-positive vulvar tumors (MIR451A) is marked with a red dot. DE values were calculated with respect to normal tissues and filtered (FC ≥ 2.0, *p*adj ≤ 0.1; see Materials and Methods). For DE values of individual miRNAs, see Table S3. Venn diagrams were created using InteractiVenn [79].

We compared miRNA expression between tumor and normal tissues of the same locations, identifying miRNAs which exhibited differential expression (DE). This type of comparison is particularly suitable for pointing out the miRNAs whose deregulation is linked with carcinogenesis. Figure 2a shows the total numbers of deregulated miRNAs in the seven tumor types differing in anatomical location and HPV status. The total numbers of deregulated miRNAs varied greatly among tumor types. Cervical HPV-positive tumors exhibited the highest number of deregulated miRNAs, followed by vulvar HPV-negative tumors. This is in accordance with the marked separation of the miRNA expression profiles of these tumor samples from all other tissues as seen in Figure 1. On the opposite end of the spectrum of miRNA deregulation, HPV-positive vulvar tumors yielded only a single downregulated miRNA (see below). Again, this was mirrored in the clustering of miRNA expression profiles; 2/3 of vulvar HPV-positive tumor samples were intermingled between normal vulvar tissues (Figure 1).

The cross-tumor comparison revealed that the largest proportion of deregulated miRNAs was specific to a single tumor type (149/239 and 94/210 upregulated and downregulated miRNAs, respectively; Figure 2b). The numbers of miRNAs which exhibited shared deregulation decreased with the increasing extent of common deregulation among the seven tumor types. Both upregulated and downregulated miRNAs followed this pattern (Figure 2b). Downregulated miRNAs were more abundant than upregulated miRNAs among the most widely deregulated miRNAs. Specifically, 58 vs. 68, 28 vs. 28, 4 vs. 14, and 0 vs. 6 miRNAs (upregulated vs. downregulated) exhibited shared deregulation in two, three, four, and five tumor types, respectively (Figure 2b). No miRNA was deregulated in more than five tumor types. The miRNAs whose deregulation was detected in most tumor types of the set are likely to play a conserved role in tumorigenesis. A literature search confirmed that all six miRNAs downregulated in 5/7 tumor types were determined to function as tumor suppressors in SCCs (Table 1). Notably, the sole miRNA downregulated in HPV-positive vulvar cancers (MIR451A) was present among these most widely deregulated miRNAs.

**Table 1:** The most commonly deregulated miRNAs which exhibited downregulation in 5/7 studied tumor types. All the listed miRNAs were demonstrated to act as tumor suppressor miRNA in SCCs, based on their experimentally confirmed characteristics relevant to carcinogenesis (invasiveness, proliferation).

Gene	miRNA	Downregulated in (Location/HPV Status) ^1^	Targets in SCCs	References
hsa-miR-101-3p	MIR101-1	anal/−, cervical/+, tonsillar/−, tonsillar/+, vulvar/−	CDK8, COX-2, CXCR7, EZH2, FOS, JAK2, MALAT1, TGFBR1, ZEB1	[42,43,44,45,46,47,48,49,50,51,52]
hsa-miR-10b-5p	MIR10B	anal/−, cervical/+, tonsillar/−, tonsillar/+, vulvar/−	HOXA1, IGF1R, TIAM1	[53,54,55]
hsa-miR-29c-3p	MIR29C	anal/−, cervical/+, tonsillar/−, tonsillar/+, vulvar/−	CCNE, ITGA6, LAMC2	[56,57]
hsa-miR-30a-5p	MIR30A	anal/−, cervical/+, tonsillar/−, tonsillar/+, vulvar/−	FOXD1, FZD2, MEF2D, WNT2	[58,59,60]
hsa-miR-451a	MIR451A	cervical/+, tonsillar/−, tonsillar/+, vulvar/−, vulvar/+	CDKN2D, ESDN, KIF2A, MAP3K1, PDPK1	[61,62,63,64]
hsa-miR-195-5p	MIR195	anal/+, cervical/+, tonsillar/−, tonsillar/+, vulvar/−	ARL2, BCL2, CCND1, CCND2, CDC42, DCUN1D1, HDGF, MYB, ROCK1, SMAD3, SMAD7, TRIM14, VEGF, YAP1	[65,66,67,68,69,70,71,72,73,74,75,76,77,78]

^1^ FC ≥ 2.0, *p*adj ≤ 0.1.

### 3.3. Limited Role of HPV in miRNA Deregulation

Since infection with high-risk HPV16 is a key factor which delineates tumors between and within different anatomical locations, we investigated whether infection with HPV is functionally translated into a common set of deregulated miRNAs. Core HPV miRNAs were focused on in two previous studies [19,32]; however, both analyses were limited to two groups of HPV-positive tumors (cervical and head and neck SCCs). Figure 2c shows that the miRNAs which exhibited deregulation only in HPV-positive tumors were present in minute numbers. Specifically, a single miRNA (miR-146a-5p) was upregulated exclusively in HPV-positive anal and cervical tumors, and a single miRNA (miR-210-3p) was upregulated exclusively in HPV-positive anal and tonsillar tumors. Both miRNAs have been previously shown to be involved in tumorigenesis. The deregulation of miR-146a-5p was observed in many types of cancers (reviewed in [80]), including cervical tumors where it functions as oncomiR, stimulating proliferation of cells [81]. MiR-210-3p was characterized as a hypoxia-regulated miRNA involved in many biological processes and was shown to be overexpressed in many tumors including head and neck cancer [82]. Five miRNAs (miR-663a, miR-769-5p, miR-1307-5p, miR-3196, miR-4800-3p) were upregulated and three miRNAs (miR-139-5p, miR-142-5p, miR-574-3p) were downregulated exclusively in HPV-positive tonsillar and cervical tumors. Downregulation of miR-139-5p and miR-574-3p in HPV-positive tonsillar and cervical tumors is in agreement with the results reported by Lajer et al. [19]. The deregulation of both miRNAs was observed in many other cancer types; the role of miR-139-5p as a tumor suppressor was confirmed in cervical tumors and head and neck tumors [83,84]; however, the function of miR-574-3p was not studied in these types of tumors. No miRNA was deregulated in more than two types of exclusively HPV-positive tumors (Figure 2c, Table S3). Therefore, our results do not support the existence of universal “Core HPV” miRNAs whose deregulation is dependent on HPV infection.

In our set of seven SCC types, the highest similarity in miRNA deregulation was detected between cervical and HPV-negative vulvar tumors. These tumors exhibited the largest total numbers of deregulated miRNAs (Figure 2a). Of all the miRNAs deregulated in HPV-negative vulvar tumors, 69% and 76% (upregulated and downregulated miRNAs, respectively) were also deregulated in cervical tumors. Twenty-nine upregulated miRNAs and 42 downregulated miRNAs exhibited exclusive deregulation in both cervical and vulvar HPV-negative tumors (Figure 2c). For these miRNAs, we mined available literature for experimental evidence of their role in carcinogenesis of SCCs, i.e., of the effect on cell proliferation and invasiveness. While information was lacking for most upregulated miRNAs, 81% of downregulated miRNAs were experimentally characterized (Table 2). Of these 34 miRNAs, 30 were determined to function as tumor suppressors (17—in cervical carcinomas, 13—in other types of SCCs); their downregulation observed here is thus in line with their carcinogenic role in SCCs. Since the widespread downregulation of tumor suppressor miRNAs in vulvar HPV-negative tumors is independent of HPV infection, it should be considered convergent with cervical tumors.

Unless diagnosed early, cervical cancers are notorious for their poor prognosis; the otherwise high mortality is kept in check only by strenuous screening and HPV vaccination. In our set of SCCs, cervical carcinomas exhibited the most extensive miRNA deregulation (Figure 2a). Among vulvar carcinomas, the prognosis (survival rate) of HPV-negative tumors is far worse than that of HPV-positive tumors [85,86]; due to additional differences other than HPV status, both types of vulvar carcinomas are regarded as separate rather than related cancer types [87]. With respect to the total extent of miRNA deregulation, vulvar SCCs with differing HPV status are fundamentally different (extensive vs. negligible deregulation; Figure 2a); our findings thus tentatively suggest that the pattern of miRNA deregulation might be linked to the severity of gynecological malignancies. The current understanding of molecular mechanisms of vulvar carcinogenesis is extremely limited [88]. The downregulation of multiple tumor suppressor miRNAs detected in this study suggests a possible mechanism contributing to a severe course of vulvar HPV-negative cancers. Interestingly, differences in the prognosis [33] and miRNA deregulation pattern [32] were also previously found between HPV-positive and HPV-negative tonsillar tumors, although their potential connection has not been investigated yet.

**Table 2:** MicroRNAs exclusively downregulated in both cervical HPV-positive and vulvar HPV-negative tumors. The designation of individual miRNAs as tumor suppressors or oncogenes is based on their experimentally confirmed characteristics relevant to carcinogenesis (invasiveness, proliferation). Literature mining was aimed primarily at studies on cervical cancers; if none were available, experimental evidence for other types of SCCs was collected.

Gene	miRNA	Cervical Cancer		Vulvar HPV-Negative Cancer		Function in SCCs	SCC Type ^1^	Targets	References
		FC of Expression	*p*adj	FC of Expression	*p*adj				
hsa-miR-103b	MIR103B1	16.8	1.2 × 10^−4^	11.3	4.7 × 10^−3^	Tumor suppressor	OSCC	SALL4	[89]
hsa-miR-107	MIR107	15.8	8.6 × 10^−3^	12.9	1.5 × 10^−2^	Tumor suppressor	CC	MCL1	[90]
hsa-miR-125b-5p	MIR125B1	6.4	1.8 × 10^−3^	14.6	3.7 × 10^−7^	Tumor suppressor	CC	PIK3CD	[91]
hsa-miR-136-3p	MIR136	31.6	1.0 × 10^−7^	6.5	1.1 × 10^−2^	Tumor suppressor	CC	E2F1	[92]
hsa-miR-148a-3p	MIR148A	22.4	3.2 × 10^−8^	13.3	4.7 × 10^−5^	Tumor suppressor	ESCC, OSCC	MAP3K9, IGF1R	[93,94]
hsa-miR-148b-3p	MIR148B	10.4	6.7 × 10^−3^	10.4	3.2 × 10^−2^	Tumor suppressor	CC	CASP3	[95]
hsa-miR-152-3p	MIR152	11.8	8.8 × 10^−5^	3.7	6.8 × 10^−2^	Tumor suppressor	CC	KLF5	[96]
hsa-miR-16-5p	MIR16-1	7.2	3.8 × 10^-3^	10.8	1.8 × 10^−4^	Tumor suppressor	OSCC	AKT3, BCL2L2	[97]
hsa-miR-17-5p	MIR17	22.3	6.4 × 10^−3^	11.0	3.7 × 10^−2^	Tumor suppressor	CC	TP53INP1	[98]
hsa-miR-199a-3p	MIR199A1	38.2	4.4 × 10^−9^	10.7	9.3 × 10^−4^	Tumor suppressor	HNSCC	ITGA3	[99]
hsa-miR-199a-5p	MIR199A1	4.8	2.0 × 10^−3^	4.5	1.2 × 10^−2^	Tumor suppressor	HNSCC, OSCC	SOX4, IKK2, ITGA3	[99,100,101]
hsa-miR-199b-3p	MIR199B	34.1	7.8 × 10^−9^	10.6	9.3 × 10^−4^	Tumor suppressor	HNSCC	ITGA3	[99]
hsa-miR-199b-5p	MIR199B	13.1	3.2 × 10^−8^	9.3	3.2 × 10^−4^	Tumor suppressor	HNSCC	ITGA3	[99]
hsa-miR-211-5p	MIR211	15.6	3.0 × 10^−2^	58.2	2.4 × 10^−5^	Tumor suppressor	CC	SPARC	[102]
hsa-miR-218-5p	MIR218-1	11.4	1.2 × 10^−3^	4.3	9.3 × 10^−2^	Tumor suppressor	CC	IDO1	[103]
hsa-miR-222-3p	MIR222	5.7	7.8 × 10^−4^	3.8	2.0 × 10^−2^	Tumor suppressor	CC	ALDH1	[104]
hsa-miR-23a-3p	MIR23A	3.7	1.9 × 10^−2^	3.2	6.8 × 10^−2^	Tumor suppressor	OSCC	FGF2	[105]
hsa-miR-24-3p	MIR24-1	7.5	3.0 × 10^−4^	9.9	9.3 × 10^−4^	Tumor suppressor	LSCC	S100A8, XIAP	[106,107]
hsa-miR-27a-3p	MIR27A	4.0	6.0 × 10^−3^	3.2	4.5 × 10^−2^	Tumor suppressor	CC	TGFBRI	[108]
hsa-miR-27b-3p	MIR27B	5.5	2.9 × 10^−3^	6.8	1.3 × 10^−3^	Tumor suppressor	ESCC	NFE2L2	[109]
hsa-miR-30d-5p	MIR30D	2.4	7.7 × 10^−2^	2.7	7.6 × 10^−2^	Tumor suppressor	ESCC	EZH2	[110]
hsa-miR-33a-5p	MIR33A	6.0	3.6 × 10^−2^	12.2	2.8 × 10^−2^	Tumor suppressor	CC	TWIST1	[111]
hsa-miR-376c-3p	MIR376C	45.4	9.0 × 10^−6^	12.8	1.8 × 10^−2^	Tumor suppressor	CC	BMI1	[112]
hsa-miR-411-5p	MIR411	67.3	4.1 × 10^−5^	8.9	6.6 × 10^−2^	Tumor suppressor	CC	STAT3	[113]
hsa-miR-99a-3p	MIR99A	12.5	6.7 × 10^−4^	26.9	1.1 × 10^−3^	Tumor suppressor	CC	TRIB2	[114]
hsa-miR-99a-5p	MIR99A	4.2	4.4 × 10^−2^	17.7	3.7 × 10^−7^	Tumor suppressor	CC	TRIB2	[114]
hsa-let-7a-5p	MIRLET7A1	9.5	2.5 × 10^−4^	9.0	9.3 × 10^−4^	Tumor suppressor	CC	PKM2, TGFBR1	[115,116]
hsa-let-7b-5p	MIRLET7B	4.3	2.4 × 10^−3^	2.7	7.7 × 10^−2^	Tumor suppressor	CC	KIAA1377	[117]
hsa-let-7c-5p	MIRLET7C	14.0	4.0 × 10^−5^	11.8	2.4 × 10^−5^	Tumor suppressor	ESCC, HNSCC	CTHRC1, IGF1R, HMGA2	[118,119]
hsa-let-7e-5p	MIRLET7E	19.3	3.4 × 10^−4^	5.8	3.7 × 10^−2^	Tumor suppressor	HNSCC	CCR7	[120]
hsa-miR-106b-5p	MIR106B	6.2	3.8 × 10^−2^	8.4	3.0 × 10^−2^	oncogene	CC	DAB2	[121]
hsa-miR-130a-3p	MIR130A	37.4	1.6 × 10^−8^	8.4	6.9 × 10^−3^	oncogene	CC	RUNX3	[122]
hsa-miR-141-3p	MIR141	3.7	3.8 × 10^−2^	3.1	6.9 × 10^−2^	oncogene	CC	FOXA2	[123]
hsa-miR-4454	MIR4454	7.8	3.3 × 10^−4^	5.2	2.6 × 10^−2^	oncogene	CC	ABHD2, NUDT21	[124]
hsa-miR-191-5p	MIR191	8.0	5.2 × 10^−5^	5.8	2.8 × 10^−3^	n.d.			
hsa-miR-3074-5p	MIR3074	7.5	3.0 × 10^−4^	9.9	9.3 × 10^−4^	n.d.			
hsa-miR-3195	MIR3195	3.0	9.2 × 10^−2^	3.6	5.7 × 10^−2^	n.d.			
hsa-miR-4286	MIR4286	5.2	5.1 × 10^−2^	6.0	4.1 × 10^−2^	n.d.			
hsa-miR-6510-3p	MIR6510	10.7	4.3 × 10^−2^	13.4	1.9 × 10^−2^	n.d.			
hsa-miR-660-5p	MIR660	20.0	5.7 × 10^−4^	7.1	3.4 × 10^−2^	n.d.			
hsa-miR-887-3p	MIR887	8.8	1.7 × 10^−3^	4.2	3.5 × 10^−2^	n.d.			
hsa-let-7f-5p	MIRLET7F1	7.4	2.8 × 10^−4^	5.0	3.1 × 10^−3^	n.d.			

^1^ CC: cervical carcinoma; ESCC: esophageal SCC; HNSCC: head and neck SCC; LSCC: laryngeal SCC; OSCC: oral SCC; n.d.: not determined (no experimental evidence for SCC available).

In conclusion, this study demonstrates that miRNA deregulation in various SCC types is largely tumor-specific and that the HPV status of SCCs is not a determinant of either the extent or the composition of the deregulated miRNA pool. Our findings may provide useful directions for further studies on the pathogenesis of individual types of SCCs with respect to their differences in anatomical location and HPV status.

## Data Availability

Data supporting the presented results are available in three supplementary tables. Sequencing reads were deposited in the SRA, GenBank (BioProject ID: PRJNA718204).

## Supplementary Materials

The following are available online at https://www.mdpi.com/article/10.3390/biom11050764/s1, Table S1: Yields of sequencing reads in sample tissues, Table S2: Relative expression of miRNAs in sample tissues, Table S3: Differentially expressed miRNAs in different tumor types.
